# QS molecules change the planktonic/mineral subpopulations distribution of moderately thermophilic leaching bacteria in pyrite and decrease leaching in chalcopyrite

**DOI:** 10.3389/fmicb.2025.1592588

**Published:** 2025-05-16

**Authors:** Beatriz Salas, Sören Bellenberg, Emelie Nilsson, Luna López-Tomasovic, Mark Dopson, Mario Vera

**Affiliations:** ^1^Department of Hydraulic & Environmental Engineering, Pontificia Universidad Católica de Chile, Santiago, Chile; ^2^Institute for Biological and Medical Engineering, Schools of Engineering, Medicine & Biological Sciences, Pontificia Universidad Católica de Chile, Santiago, Chile; ^3^Centre for Ecology and Evolution in Microbial Model Systems (EEMiS), Linnaeus University, Kalmar, Sweden; ^4^Department of Mining Engineering, School of Engineering, Pontificia Universidad Católica de Chile, Santiago, Chile

**Keywords:** quorum sensing, bioleaching, moderately thermophilic bacteria, metal sulfides, transcriptomics

## Abstract

Biomining is a sustainable alternative to conventional mineral processing that uses acidophilic microorganisms to catalyze the extraction of valuable metals from sulfide minerals. Mixed microbial consortia composed of moderate thermophiles such as *Sulfobacillus* and some *Leptospirillum* species improve metal extraction efficiency at higher temperatures compared to pure cultures of mesophiles. However, quorum sensing (QS), which regulates microbial interactions and likely influences bioleaching performance, has not been studied in these species. In this study, treatment of a moderately thermophilic biomining consortium with QS compounds, termed diffusible signal factors (DSF), reduced pyrite and chalcopyrite dissolution via an inhibitory effect on iron oxidation and mineral colonization by the mixed culture. Furthermore, QS molecules changed the distribution of planktonic/mineral subpopulations of the acidophilic species. In addition, DSF compounds induced *Acidithiobacillus caldus* motility and dispersion from pyrite with a concomitant expansion of *Leptospirillum ferriphilum* on the mineral surface while in contrast, the acyl-homoserine lactone mediated QS system repressed *L. ferriphilum* motility. Moreover, the addition of QS molecules induced a second response related to the detrimental effect of high concentrations of fatty acids on cells, with an activation of detoxification mechanisms. Overall, QS regulated key target microbial interactions that opens the possibility to improve chalcopyrite bioleaching in the studied consortia.

## 1 Introduction

Biomining is an advantageous alternative to conventional sulfide mineral processing due to lower energy consumption, mild reaction conditions that represent a lower environmental impact, and the ability to extract metals from low-grade ores. Bioleaching, a type of biomining whereby the target metal is part of the mineral matrix, has been applied to metal sulfides (MS) to solubilize and recover valuable metals such as copper, cobalt, nickel, and zinc (Vera et al., 2022). Bioleaching environments present challenging conditions with highly acidic pH values and with a wide range of temperatures that select for extremely acidophilic bacteria that have an optimum pH for growth of ≤3 (Rawlings and Johnson, 2007). Many of these microorganisms are able to oxidize ferrous iron and/or inorganic sulfur compounds (ISC) and as a consequence, to catalyze the dissolution of minerals (Johnson and Quatrini, 2020). Among other environmental factors, temperature determines the diversity of microorganisms present in bioleaching communities. As an option to overcome the slow rate of metal extraction during bioleaching, it is proposed to use moderate thermophiles (MT) instead of mesophilic microorganisms due to their accelerated metabolic activity that will increase reaction kinetics and facilitate metal dissolution (Brierley and Brierley, 1986; Pradhan et al., 2008). MT bacteria present a balance between metabolic activity and process feasibility as while using thermophiles (optimal growth >60°C) theoretically would further increase the reaction rates. However, several technical and economic considerations preclude their use such as industrial-scale bioleaching setups (heaps, dumps) are difficult to heat and maintain at >60°C. Therefore, moderate thermophiles are more widely investigated and used to improve sulfide ore dissolution rates (Rhodes et al., 1998; Marhual et al., 2008; Wang et al., 2014; Huang et al., 2017).

MT are typically prevalent in the core of bioheaps where the temperature can be 10–15°C higher than ambient (Das et al., 1999). Under these conditions (40–60°C), *Acidithiobacillus caldus* (*A. caldus*) and *Leptospirillum ferriphilum* (*L. ferriphilum*) dominate along with species from the genera *Alicyclobacillus*, *Sulfobacillus*, *Ferrimicrobium*, *Acidimicrobium*, *Ferrithrix*, and proposed genus “*Acidithiomicrobium*” (Quatrini and Johnson, 2016). *A. caldus* is an aerobic, ISC oxidizing obligate chemolithoautotroph (Hallberg and Lindström, 1994; Acuña et al., 2013) with a higher optimal growth temperature to most members of *Acidithiobacillus* genus at 45°C. Recently this species has been proposed to belong to the novel proposed genus “*Fervidacidithiobacillus*” (Moya-Beltrán et al., 2021). Importantly, *A. caldus* is suggested to be the primary sulfur oxidizer in mineral concentrate reactors operating at temperatures above 40°C (Dopson and Lindström, 1999). *L. ferriphilum* is the single moderately thermophilic *Leptospirillum* species and uses iron(II) as sole energy source and fixes carbon dioxide (Hippe, 2000; Christel et al., 2017). With a pH tolerance range of 1.3–2.0, *Leptospirillum* spp. are distributed in natural and industrial environments where the accelerated oxidation of sulfide ores creates acidic, metal-rich ecosystems (Schippers et al., 2014). Although *L. ferriphilum* is the only recognized *Leptospirillum* species with a temperature optimum of 37°C, many isolated strains are defined as being moderately thermophilic (Dopson and Johnson, 2012; Vardanyan et al., 2023; Casas-Vargas et al., 2024). The *Sulfobacillus* genus is composed by species able to use elemental sulfur, ISC, and iron(II) ions as energy sources under aerobic chemolithoautotrophic or mixotrophic conditions (Golovacheva and Karavaiko, 1978; Karavaiko et al., 1988; Norris et al., 1996). Moderately thermophilic communities include *S. thermosulfidooxidans*, which has optimum growth conditions of approximately 50–55°C with a pH of 1.9–2.4 (Vos et al., 2011). This species may increase bioleaching activity, especially in sulfur-rich acidic environments at elevated temperatures where sulfur-oxidizing mesophilic bacteria cannot grow (Dopson and Lindström, 2004).

Microbial interactions are widespread in bioleaching systems and mixed cultures are typically more robust and efficient than pure cultures in catalyzing sulfide mineral oxidation. For instance, associations of leaching microorganisms, and especially indigenous leaching consortia, are more stable and efficient for use in commercial bioleaching installations (Johnson, 2001; Falco et al., 2003; Akcil et al., 2007; Rawlings and Johnson, 2007). Furthermore, microbial interactions could have significant effects on microbial consortium dynamics and potentially influence bioleaching performance (Johnson, 1998; Erlich, 1999; Johnson, 2001; Bellenberg et al., 2014; Bellenberg et al., 2018; Christel et al., 2018). Therefore, using bioleaching consortia can improve the extraction rate of valuable minerals over pure cultures and current efforts should be addressed to their study.

Quorum sensing (QS) is one of the main bacterial communication systems by which they monitor their population density and regulate gene expression by the release and detection of small molecules called autoinducers (AIs), which mediate intracellular signaling, leading to several adaptive responses (Camilli and Bassler, 2006; Boyer and Wisniewski-Dyé, 2009). Two types of QS have been reported in acidophilic bioleaching microorganisms. Some *Acidithiobacillus* and *Acidiferrobacter* species possess a QS system mediated by acyl-homoserine lactones (AHLs) (Farah et al., 2005; Ruiz et al., 2008; González et al., 2013; Bellenberg et al., 2014; Díaz et al., 2020; Gao et al., 2021). In addition, *L. ferriphilum* DSM 14647^T^ and *L. ferrooxidans* C2-3 species produce and use the diffusible signaling factors (DSF) QS system (Christel et al., 2017; Bellenberg et al., 2018; Buetti-Dinh et al., 2020; Bellenberg et al., 2021). In some cases, AI production is associated with inhibitory effects on oxidizing activity and/or pyrite leaching, changes in biofilm formation patterns, and exopolysaccharide (EPS) production (Bellenberg et al., 2014; Bellenberg et al., 2018; Bellenberg et al., 2021). Recently, a decrease in mineral-attached populations of *A. caldus*, *L. ferriphilum*, and *S. thermosulfidooxidans* was demonstrated after DSF addition (Bellenberg et al., 2018). Furthermore, external addition of *cis*-11-methyl-2-dodecenoic acid and/or cis-2-dodecenoic acid (BDSF) inhibits growth and the ability to oxidize iron(II)-ions in several acidophilic leaching species, including *L. ferriphilum*^T^. Finally, DSF molecules produce a toxic effect in *L. ferriphilum* due to expression of detoxification systems such as multidrug efflux pumps, probably to extrude these molecules (Bellenberg et al., 2021). However, due to the use of axenic cultures, data on the effect of QS on bioleaching systems is very limited and most previous studies neglected the relationships between bioleaching performance, microbial consortium dynamics, and the presence of QS molecules.

This work focused on MT bacteria relevant in bioleaching operations that play important roles in sulfide mineral dissolution (Sand and Gehrke, 2006; Gautier et al., 2008). An MT consortium was designed that was composed of *L. ferriphilum*^T^, *S. thermosulfidooxidans*^T^, and *A. caldus*^T^. These species were selected to complement their metabolic properties including ferrous iron (*L. ferriphilum* and *S. thermosulfidooxidans*) and ISC (*A. caldus* and *S. thermosulfidooxidans*) oxidizing capacities as well as autotrophy (*A. caldus* and *L. ferriphilum*) and mixotrophy (*S. thermosulfidooxidans*). This study investigated the effects of QS signaling on iron oxidation plus consortium dynamics and to correlate these parameters with the development and establishment of a microbial community for copper extraction. Verifying a QS inhibitory effect on iron oxidation and mineral colonization of the MT consortium and describing the changes in the community composition will reveal key microbial interactions that opens the possibility to improve pyrite bioleaching in the studied consortium.

## 2 Materials and methods

### 2.1 Bacterial strains and growth conditions

The utilized species were *L. ferriphilum* DSM 14647^T^, *A. caldus* DSM 8584^T^, and *S. thermosulfidooxidans* DSM 9293^T^. *L. ferriphilum* was grown in Mackintosh basal salt solution (Mac medium) pH 2.0 (Mackintosh, 1978) with 72 mM iron(II) ions supplied as FeSO_4_·7H_2_O. *A. caldus* was grown in pH 2.5 Mac medium with 1% (wt/vol) elemental sulfur. For *S. thermosulfidooxidans* DSM 9293^T^, Mac medium pH 2.5 was amended with 34 mM iron(II) ions, 1% (wt/vol) elemental sulfur, and 0.02% (wt/vol) yeast extract. Mac medium and yeast extract were autoclaved separately at 121°C for 20 min. Elemental sulfur was autoclaved at 110°C for 90 min. Ferrous sulfate solution (3.6 M FeSO_4_·7H_2_O, pH 1.2) was sterilized with a 0.22 μm pore (Millipore) filter. Cultures were incubated at 40°C under constant shaking at 140 rpm.

### 2.2 Quorum sensing signal compounds

Mineral cultures were amended as specified with QS signal compounds directly before inoculation or 24 h after inoculation. The DSF-family signal compounds DSF or (Z)-11-methyl-2-dodecenoic acid (CAS 677354-23-3; Sigma) and BDSF or (Z)-2-dodecenoic acid (CAS 55928-65-9; Sigma) were used in combination at 2 μM per compound. N-acyl-DL-homoserine lactones (AHLs) were used as a mixture of N-dodecanoyl-DL-homoserine lactone (C12-AHL, CAS 8627-38-8, Sigma), N-tetradecanoyl-DL-homoserine lactone (C14-AHL, CAS 98206-80-5, Sigma), N-(3-hydroxydodecanoyl)-DL-homoserine (3-OH-C12-AHL, CAS 182359-60-0, Sigma), and N-(3-hydroxytetradecanoyl)-DL-homoserine lactone (3-OH-C14-AHL, CAS 172670-99-4, Sigma) at 5 μM per compound. Stock solutions of these compounds were prepared in dimethyl sulfoxide (DMSO) at 50 mM for AHL molecules and 5 mM in the case of DSF-family signal compounds. Consequently, control assays were amended with equal volumes of DMSO; 6 mM and 11.3 mM, respectively.

### 2.3 Preparation of pyrite and chalcopyrite

Both minerals were crushed using a vibratory pulverizer and fractioned in particle sizes between 50 and 100 μm. Pyrite (Peru) was characterized by digestion for 1 h with four different acids (HCl, H_2_SO_4_, HNO_3_, and HF) and the solution analyzed by atomic absorption spectroscopy (AAS) in a PinAacle 900F spectrometer (PerkinElmer, Shelton, CT, United States). The iron content of pyrite was 46% (wt/wt). Chalcopyrite grains were obtained from a flotation concentrate provided by Boliden AB mine (Sweden). It possesses a purity exceeding 98% through aqua regia digestion and containing 29.5% (wt/wt) copper. Pyrite grains were sterilized as previously described (Bellenberg et al., 2021). Briefly, 100 g pyrite grains were boiled in 200 mL 6 M HCl for 30 min and then washed with deionized water until the pH was neutral. Afterwards, grains were stirred twice in 100 mL acetone for 30 min to remove soluble sulfur compounds by discarding the solvent after the treatment. Acetone residues in the pyrite samples were evaporated in a fume hood at room temperature for 12 h, and heat-sterilized for 12 h at 120°C. Chalcopyrite sterilization procedures were carried out following a protocol published by Christel et al. (2018). Briefly, the grains were washed twice for 30 min in 10 volumes of washing solution (0.1 M EDTA, 0.4 M NaOH) to eliminate iron and copper compounds, surface elemental sulfur was removed with acetone. Finally, the mineral was dried at 60°C overnight and then sterilized at 120°C for 10 h under a nitrogen atmosphere to ensure no changes in the mineral structure occurred.

### 2.4 Preparation of mineral cultures with MT mock consortium for bioleaching assays

The MT consortium bioleaching assays were performed at 40°C in Erlenmeyer flasks with Mac medium pH 2.0 and 2% (wt/vol) pyrite or chalcopyrite grains plus 34 mM iron(II) ions. Mineral cultures were prepared in triplicate (*n* = 3) for each type of assay. Mineral dissolution and colonization assays were conducted in 250 mL-Erlenmeyer flasks with 100 mL of medium inoculated with a mixture of the three species each at 2 × 10^7^ cells/mL. DSF-signal compounds were added directly along with the inoculation. In addition, axenic cultures of the three species with pyrite as electron donor were inoculated with 5 × 10^7^ cells/mL and maintained under the same conditions for assessing pyrite bioleaching and colonization by single cultures.

Development of planktonic and mineral attached cell subpopulations of the MT consortium was conducted in dedicated cultures for each time-point for DNA extraction. Mineral cultures were prepared in 100 mL for AHLs or 200 mL for DSF/BDSF treatment and inoculated with 10^7^ cells/mL per species. QS signal compounds were added 24 h after inoculation (sampling time 1 day) and maintained for 7 and 14 days, when planktonic and mineral-attached cells were captured for DNA extraction.

RNA transcript sequencing was performed in pyrite grown cultures inoculated with 10^7^ cells/mL per species in 250 mL-Erlenmeyer flasks with 100 mL Mac medium and 2% (wt/vol) pyrite grains as sole substrate. No additional iron was added to allow the cells to grow only with the iron provided by the pyrite to avoid the presence of precipitates that could affect the RNA extraction and its stability. QS signal compounds were added 24 h after inoculation (sampling time: 1 day) and maintained for 14 days, when planktonic cells were captured for RNA extraction by centrifugation.

### 2.5 Monitoring of bioleaching experiments

The MT consortium bioleaching assays were monitored testing pH (VWR pHenomenal^™^ pH1000L, SI Analytics BlueLine 15pH probe) and redox potential (VWR pHenomenal^™^ pH1100L, Mettler Toledo InLab Redox-L), and addition to photometric determination of total copper concentration (Debernardi et al., 2012) using a plate reader (FLUOstar Omega^™^, BMG Labtech^®^). Cell counts were performed with a Thoma counting chamber on a Zeiss microscope.

### 2.6 Extraction of DNA and RNA from pyrite cultures

DNA was extracted from planktonic and pyrite-attached/biofilm cells while RNA was only extracted from planktonic cells. Culture flasks were rapidly cooled on ice and by addition of 1 volume ice-cold Mac medium (pH 1.8). For planktonic cell samples the cultures (*n* = 4 for RNA, and *n* = *3* for DNA extraction) were centrifuged at 12,500 × g for 10 min at 4°C. The resulting cell pellet was washed twice by re-suspending in 2 mL of sterile, ice-cold Mac medium (pH 1.8), and then flash frozen in liquid nitrogen. Meanwhile, the pyrite mineral pellet (2–4 g) was washed with ice-cold Mac medium (pH 1.8), decanted, and flash frozen in liquid nitrogen. All cell samples were stored at −80°C until extraction with RNeasy^®^ PowerSoil^®^ Total RNA Kit (RNA) in combination with RNeasy^®^ PowerSoil^®^ DNA Elution Kit (DNA) as specified in the instructions except that the extraction from mineral particles was performed without using Bead Tubes. Due to the low DSF/BDSF assay biomass recovered after the treatment, captured cells were subjected to DNA extraction using hot-phenol extraction as previously described (Vera et al., 2013).

### 2.7 16S rRNA gene-based microbial community development in pyrite grown cultures

Three parallel experiments were carried out for 16S rRNA gene sequencing, although DNA was only successfully extracted from two samples of mineral attached populations from the DSF/BDSF treatments due to cell damage induced by this treatment. Partial 16S rRNA genes were amplified using primers 175F and 512R (Klindworth et al., 2013) before sequencing using an Illumina MiSeq platform with MiSeq Reagent Kit v3. Taxonomical identification was performed using the QIIME 2 platform (Bolyen et al., 2019) that includes an entire pipeline from demultiplexing steps through to taxonomical assignation (Callahan et al., 2016). The overall quality of the DNA sequencing was determined by means of a Phred quality score with reads having Phred30 values and below being discarded. The final quality controlled 16S rRNA gene sequences were used for taxonomical assignation using either the SILVA (Bokulich et al., 2018) or Greengenes (McDonald et al., 2012) databases. Statistical analyses were carried out using *car* package of R. The non-parametric Kruskal–Wallis test was used to analyze species abundances between the control and DSF/BDSF or AHLs treated groups after 7 and 14 days of incubation.

### 2.8 Transcriptomic analyses of MT consortium pyrite cultures

RNA samples were subjected to ribosomal RNA depletion using the QIAseq^®^ FastSelect^™^—5S/16S/23S kit for bacterial RNA samples. Nucleic acid quantification and quality control were assessed by agarose gel electrophoresis, NanoDrop, Qubit^™^ RNA HS Assay kit (Invitrogen^®^), and the Agilent 2100 Bioanalyzer. Libraries (12 in total) were prepared by SciLifeLab, Stockholm, Sweden using the Illumina TruSeq stranded mRNA Kit. Paired-end sequencing (2 × 151 bp) was performed on one Illumina NovaSeq6000 lane using “NovaSeqXp” workflow in “S4” mode flowcell.

RNA sequences were processed with a Bcl to FastQ conversion as performed using bcl2fastq_v2.20.0.422 from the CASAVA software suite. The quality scale used was Sanger/phred33/Illumina 1.8+ at SciLifeLab, Stockholm, Sweden. The quality of the raw sequencing reads was assessed with FastQC/MultiQC (Andrews, 2010; Ewels et al., 2016). Subsequently, adapter sequences were removed using Cutadapt/TrimGalore 0.6.1 (Martin, 2011; Krueger, 2017). Paired-end transcript reads were mapped against reference genomes annotated in NCBI database (*A. caldus*: GCF_000175575.2, *L. ferriphilum*: GCF_900198525.1, and *S. thermosulfidooxidans*: GCF_900176145.1) using Bowtie2 (Langmead and Salzberg, 2012), sorted by their genomic location using the Samtools sort function (Li, 2011), and counted using FeatureCounts of the Rsubreads package (Liao et al., 2014). Raw counts were then processed for assessment of statistically significant differential RNA transcript numbers using an approach for normalization of metatranscriptomic data that eliminates the influence of taxonomic variations from functional analysis (Klingenberg and Meinicke, 2017). Taxonomic variations were assessed by calculation of relative transcripts count per species (Supplementary Figure S1). This approach requires count data to be decomposed to normalize the organism profiles independently. Then the taxon-specific scaling of organism profiles yields normalized data arranged in a metatranscriptomic count matrix. This matrix was analyzed with DESeq2 statistical tools (Love et al., 2014) to look for any general tendencies by determination of Log_2_-fold changes (LFC) and corresponding *p*-values. The *p*-value was produced by the false discovery rate (FDR) analysis and adjusted (*p*_adj_) using the Benjamini and Hochberg’s method (Benjamini and Hochberg, 1995). Differentially expressed genes (|LFC| >1, FDR *p*_adj_ <0.05) were analyzed using the clusters of orthologous groups (COG) database (Galperin et al., 2015).

### 2.9 Microscopy sample preparation

Mineral grain particle samples were withdrawn from mineral grown cultures using a sterilized micropipette tip, washed at least three times with filtered Tris-EDTA (TE) buffer (10 mM Tris Cl, pH 8.0; 1 mM EDTA), and stored at −20°C. Cells were differentially stained by incubation of pyrite grains with 1.2 μM 4′,6-diamidino-2-phenylindole (DAPI) for 15 min. After staining, mineral grains were washed twice with filtered TE buffer and finally mounted on a glass bottom cell culture dish (15 mm, Cat. No. 801002, Nest) using a glycerol-based mounting medium (CitiFluor AF2) and 22- by 50-mm cover glass.

### 2.10 High throughput epifluorescence microscopy

Images were acquired with an inverted epifluorescence microscope (EFM) Zeiss^®^ Axio Observer Z1/7 using the 40 × objective (Zeiss Objective EC Plan-Neofluar 40×/0.75M27) and a motorized microscopy stage (Scanning Stage 130×85 mot P). Image acquisition used a Zeiss filter set 49 for DAPI-stained samples (emission peak at 465 nm) and bright-field by reflected light mode for visualization and localization of opaque mineral grains. Images were processed with the software Zeiss^®^ Zen Pro version 3.0. For massive image analysis, between 18 and 72 images were taken per sample. Images were taken in tiles (mosaic) and Z-stacking modes with a focus depth of approximately 70 layers of 0.57 μm each.

EFM image analysis was conducted following an in-house procedure based on a published protocol for automated microscopy analysis of cell colonization by acidophilic microorganisms (Rossoni et al., 2024). EFM images were manually pre-filtered and then processed using a Python-based code, which allowed for the extraction of quantitative parameters of interest. Cell counting was made using DAPI-stained images, while images recorded with bright-field by transmitted light were used to calculate the grain area. These data were used to determine colony density (colonies/mm^2^) and the average of single colony area (μm^2^). The precision and repeatability of the analyzes were evaluated by coefficient of variation (CV) determination (Supplementary Figures S2, S3) with low CV values indicating a high repeatability and a more reliable result. Statistical analyses were carried out using the *car* package in R and the non-parametric Kruskal–Wallis test was used to analyze the differences between the control and DSF/BDSF treated groups after 1 and 9 days of incubation.

## 3 Results

### 3.1 Effect of DSF/BDSF signal compounds on the MT consortium planktonic growth and iron(II) oxidation

**Figure 1:**
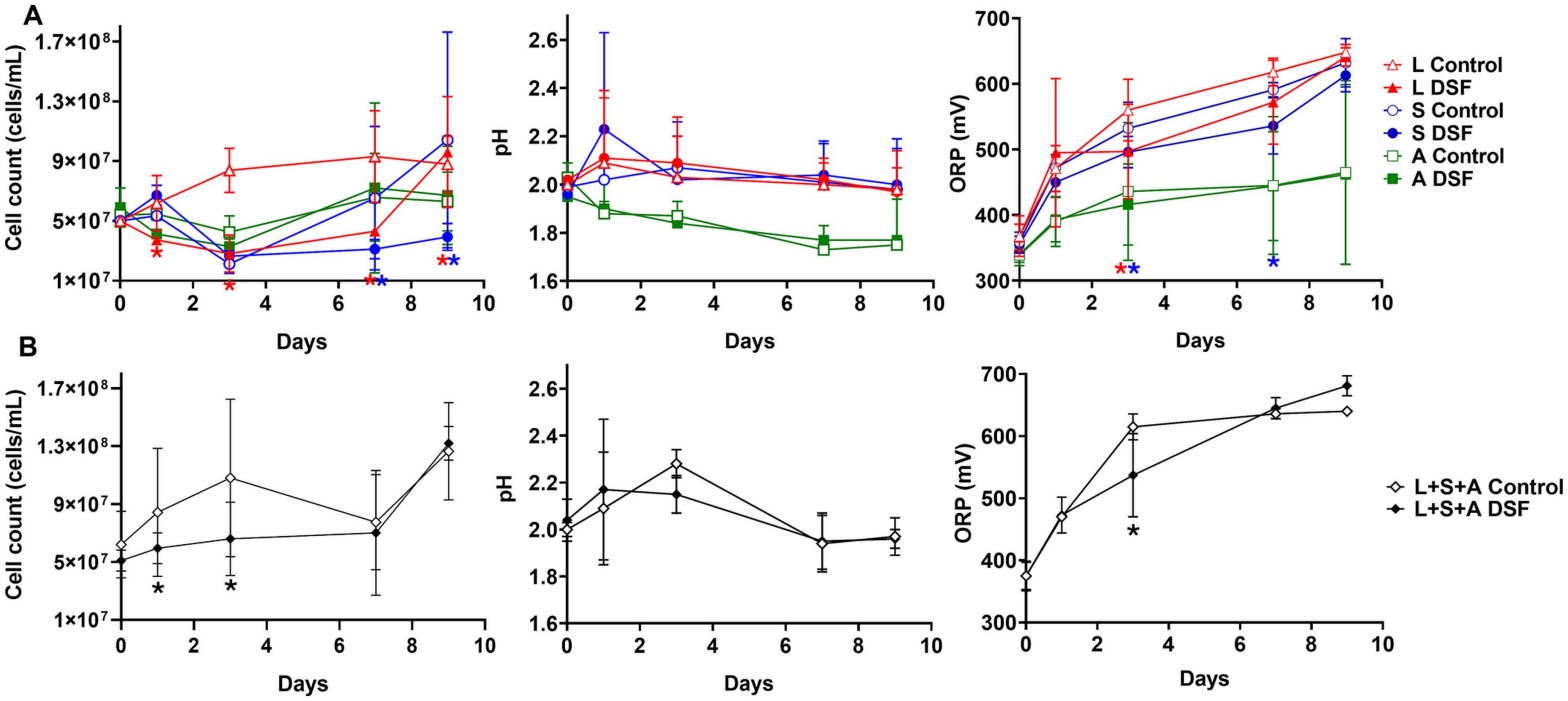
Effect of DSF/BDSF on growth and iron oxidizing activity of acidophilic leaching bacteria. Planktonic cell numbers (left), pH (middle), and redox potential (ORP) vs. Ag/AgCl (right) of axenic cultures **(A)** and MT consortium **(B)**. DSF and BDSF were added at 2 μM each and incubated with 2% (wt/vol) pyrite at 40°C and 140 rpm agitation. DSF/BDSF treated cultures are shown as filled symbols and control cultures in white symbols. Assays were performed in triplicates (*n* = 3) and significant differences are shown according to results of t-student test (*p < 0.05).

Mixed and axenic cultures were prepared using pyrite as an energy source to evaluate the effect of DSF/BDSF treatment on biomass and iron oxidizing activity (Figure 1). DSF/BDSF addition to *L. ferriphilum* produced a significant decrease in cell density from 8 × 10^7^ to 2 × 10^7^ during the first week of cultivation (*p*-value = 0.041), while a similar reduction was observed for *S. thermosulfidooxidans* for days 7 to 10 (*p* = 0.043). This was supported by significantly lower redox potential (ORP) values at 3 days for *L. ferriphilum* (*p* = 0.033) and from 3 to 7 days for *S. thermosulfidooxidans* (*p* = 0.013). In contrast, *A. caldus* cultures were unaffected by this treatment (Figure 1A) and in the case of the MT consortium, planktonic cell numbers also significantly decreased after DSF/BDSF addition during the first 7 days (*p* = 0.047). In addition, the ORP curve showed lower values between days 1 and 7 with significant values at 3 days (*p* = 0.049) after which the ORP values were similar to the control cultures (Figure 1B).

### 3.2 Effect of DSF/BDSF signal compounds on MT consortium pyrite colonization

**Figure 2:**
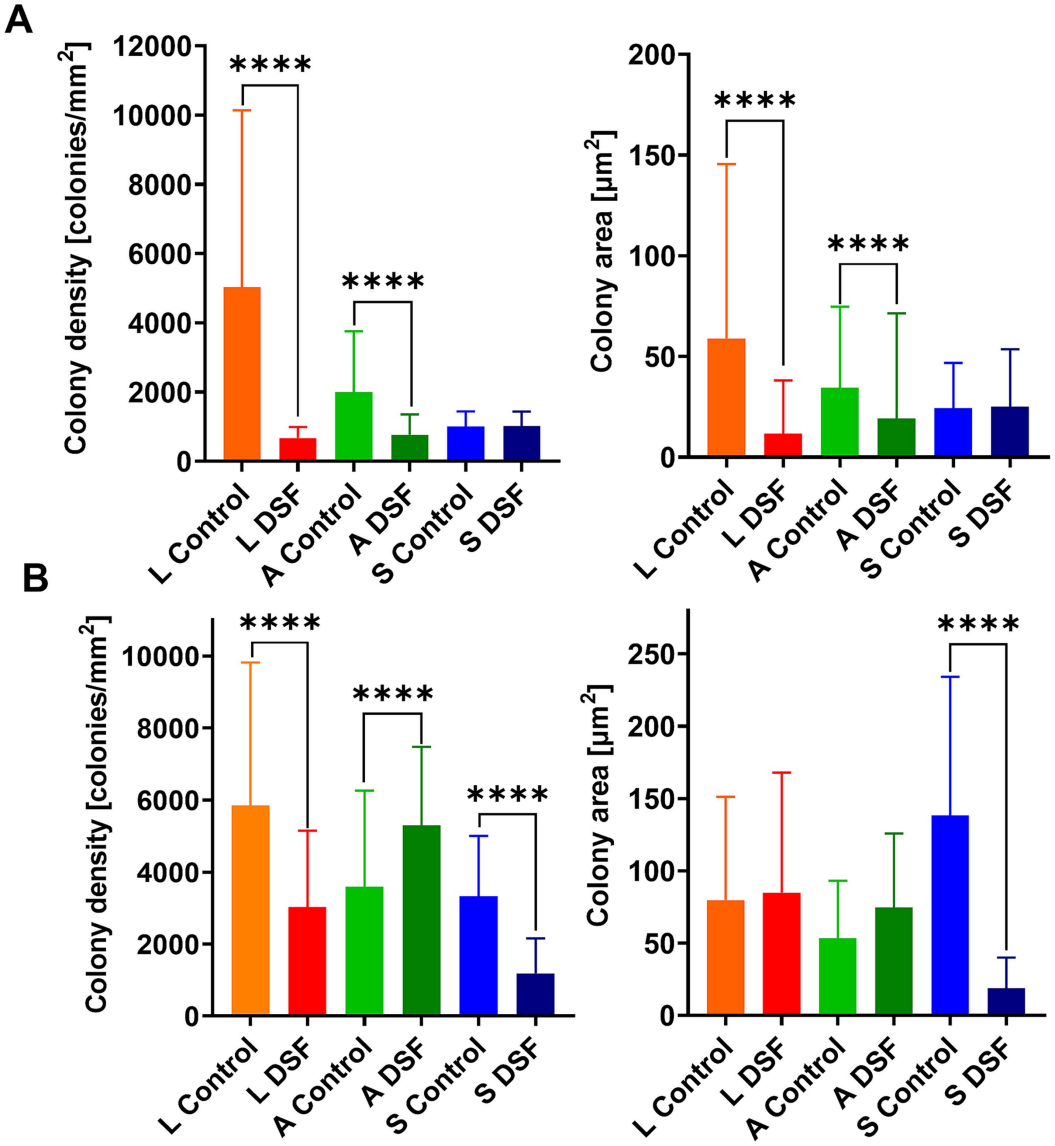
Effect of DSF/BDSF on cell attachment and biofilm formation of axenic cultures of leaching bacteria on pyrite surface. Colony density (left) and average colony area (right) were assessed for axenic cultures of *L. ferriphilum* (L), *A. caldus* **(A)**, and *S. thermosulfidooxidans* (S) at 1 **(A)** and 9 days **(B)**. DSF and BDSF were added at 2 μM each and incubated with 2% (wt/vol) pyrite at 40°C and 140 rpm agitation. Assays were duplicates (*n* = 2) and significant differences are shown according to results of Kruskal–Wallis test (^****^p < 0.00001).

**Figure 3:**
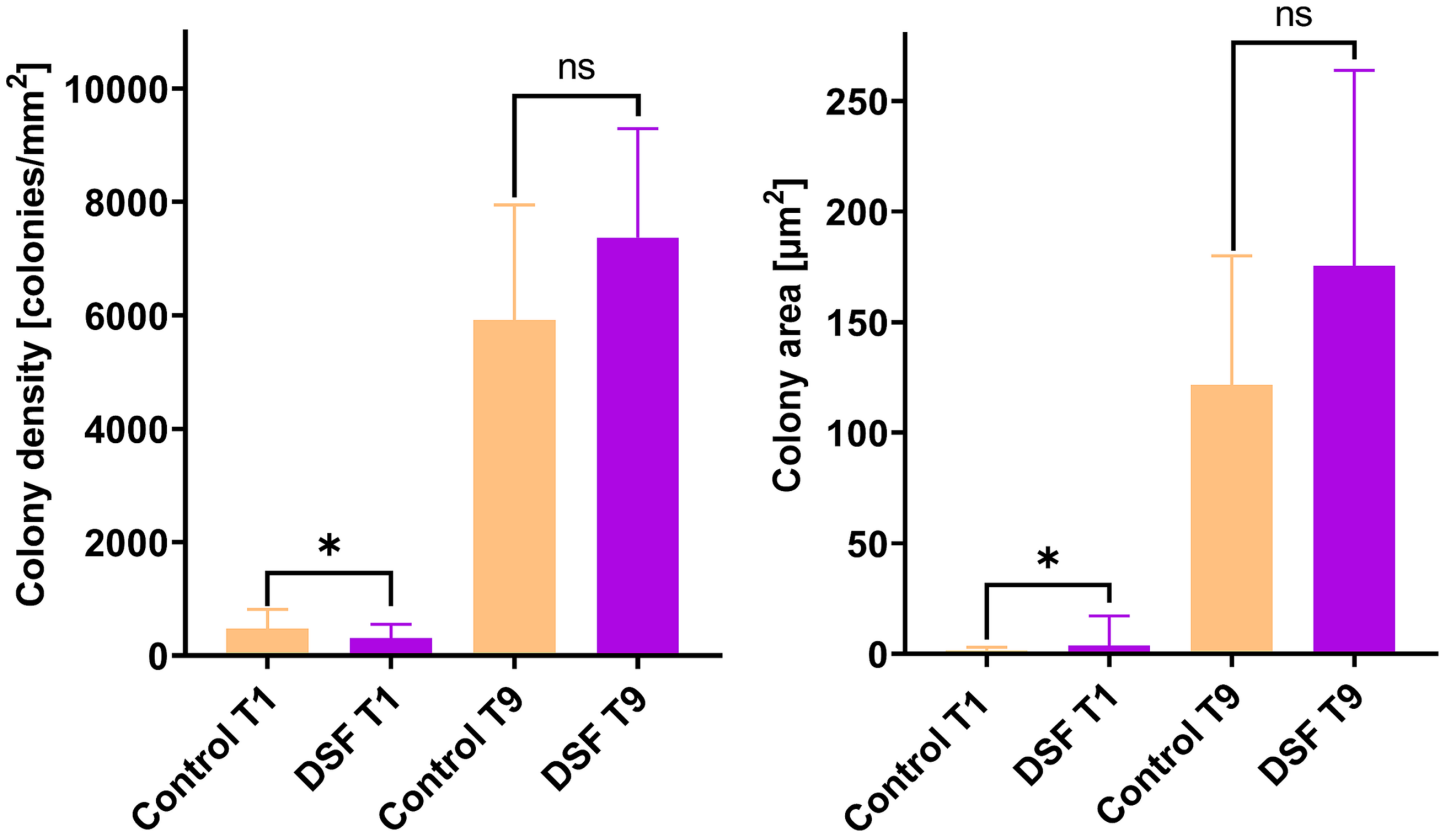
Effect of DSF/BDSF on cell attachment and biofilm formation of MT consortium on pyrite surface. Colony density (left) and average colony area (right) were assessed at 1 (T1) and 9 days (T9). DSF and BDSF were added at 2 μM each and incubated with 2% (wt/vol) pyrite at 40°C and 140 rpm agitation. Assays were duplicates (*n* = 2) and significant differences are shown according to results of Kruskal–Wallis test (^ns^*p* > 0.05 and ^*^p < 0.05).

EFM images of colonized pyrite grains after two different times during the growth of the axenic and MT consortium showed DSF/BDSF molecules addition resulted in a decrease in colony density and total colonized area in axenic cultures of *L. ferriphilum* and *A. caldus* after 1 day (*p* < 0.00005; Figure 2A). In contrast, *S. thermosulfidooxidans* did not show significant differences between the two conditions, showing low colony density and colony area in control and amended cultures, suggesting this species was unable to efficiently colonize the pyrite surface in this time frame, which is in accordance with our previous observations (Buetti-Dinh et al., 2020). After 9 days, decreased colony density was still observed for *L. ferriphilum* (Figure 2B), suggesting that although its subpopulations appeared to recover, its attachment to the pyrite surface, colony density was likely delayed by the initial detrimental effects of DSF/BDSF addition. In the case of *S. thermosulfidooxidans* after 9 days, the control cultures showed better colonization levels compared to 1 day, but this improvement was prevented when DFS/BDSF molecules were added (Figure 2B). In contrast, *A. caldus* exhibited a recovery in cell density after 9 days (Figure 2B). In addition, the MT consortium was less sensitive to DSF/BDSF addition as a reduced cell density was only observed after 1 day of treatment (*p* < 0.05), while at the end of the assay there were no significant differences between the treated consortium and control cultures (Figure 3). Finally, the MT consortium showed a better cell colonization at 9 days (~1.5 times increase on colony densities) in comparison to the *L. ferriphilum* axenic cultures (*p* < 0.05; Figure 2B).

### 3.3 Effect of DSF/BDSF signal compounds on MT consortium mediated chalcopyrite dissolution and mineral colonization

**Figure 4:**
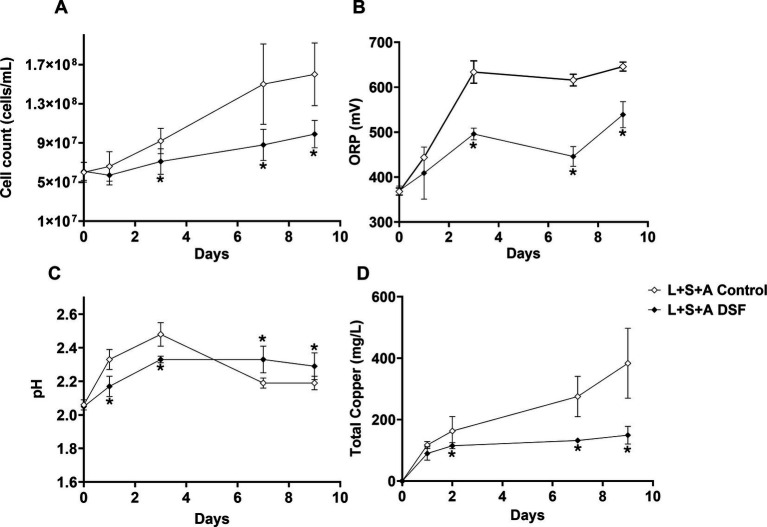
Effect of DSF/BDSF on MT consortium chalcopyrite bioleaching. Planktonic cell numbers **(A)**, redox potential (ORP) vs. Ag/AgCl **(B)**, pH **(C)**, and released copper **(D)**. DSF and BDSF were added at 2 μM each and incubated with 2% chalcopyrite at 40°C and 140 rpm agitation. DSF/BDSF treated cultures are shown in black diamonds with control cultures in white diamonds. Assays were performed in triplicates (*n* = 3) and significant differences are shown according to results of *t*-test (*p < 0.05).

**Figure 5:**
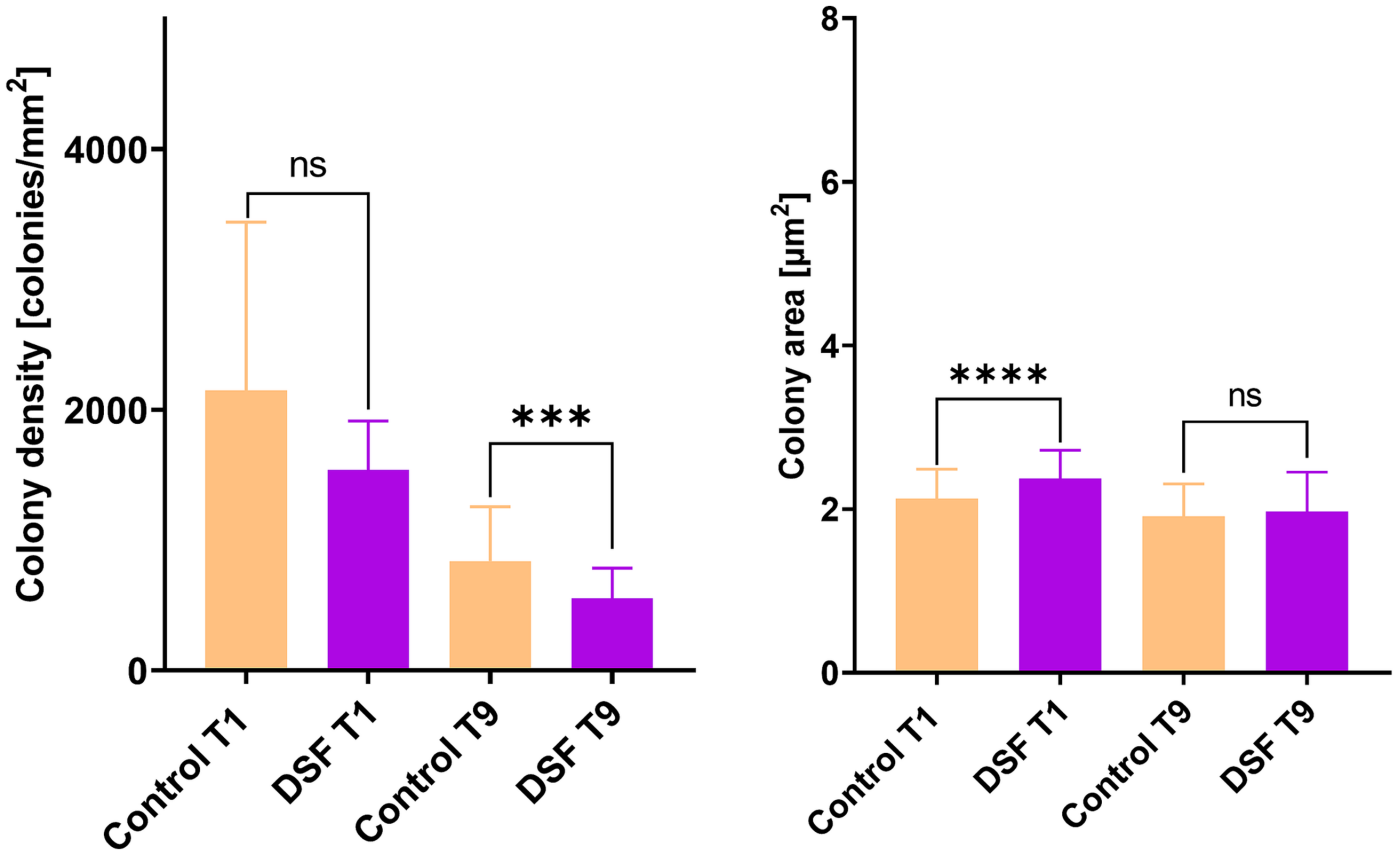
Effect of DSF/BDSF on cell attachment and biofilm formation of MT consortium in chalcopyrite. Colony density (left), average colony area (middle), and radii (right) were assessed at 1 (T1) and 9 days (T9). DSF and BDSF were added at 2 μM each and incubated with 2% (wt/vol) chalcopyrite at 40°C and 140 rpm agitation. Assays were duplicates (*n* = 2) and significant differences are shown according to results of Kruskal–Wallis test (^ns^*p* > 0.05, ^***^p < 0.0005, and ^****^p < 0.00001).

Growth and attachment assays with the MT consortium grown on 2% (wt/vol) chalcopyrite were performed to evaluate if reduced iron oxidation activity upon of DSF/BDSF addition to pyrite grown cultures was also observed with this mineral (Figure 1). The DSF/BDSF addition resulted in a decreased cell density (*p* = 0.015) and ORP (*p* = 0.013) compared to the control after 9 days (Figures 4A,B). This observation correlated with a temporal decrease of DSF and BDSF levels compared to their concentrations between days 1 and 9 (data not shown). DSF/BDSF treatment prevented pH rise during the first 5 days of cultivation compared to untreated MT consortium (*p* = 0.021), after this period the effect was insignificant (*p* = 0.051) (Figure 4C). These DSF/BDSF induced changes resulted in a lowered chalcopyrite dissolution as judged by the total copper ion concentration (*p* = 0.0186) (Figure 4D). Accordingly, DSF/BDSF presence also hindered bacterial colonization on chalcopyrite, with EFM analysis showing a significant reduction of cell density compared to the control consortium at 9 days after treatment (*p* < 0.0005; Figure 5).

Comparison of colonization levels of MT consortium control cultures between pyrite and chalcopyrite showed the colonization was significantly lower in chalcopyrite compared to pyrite at 1 and 9 days (*p* < 0.0001 for both times). For instance, comparison between pyrite and chalcopyrite colonization parameters after 9 days exhibited a colony density reduction from ~7,000 to ~1,000 colonies/mm^2^ and also a decrease in the average colony area from ~150 μm^2^ to ~2 μm^2^.

Although DSF/BDSF addition lowered ORP values toward levels suggested to favor chalcopyrite dissolution, the results indicated a reduction in chalcopyrite dissolution. This was probably due to an inhibitory effect by DSF/BDSF on the MT culture iron oxidation activity rather than a cell dispersal effect on chalcopyrite grains. This was likely as planktonic cell counts of DSF/BDSF treated cultures showed around 50% lower cell densities compared to the untreated MT consortium after 9 days (Figure 4A). Therefore, the lower colonization determined after DSF/BDSF addition cannot be explained by an increase in the planktonic subpopulation compared to the biofilm cells.

### 3.4 Effect of QS molecules on MT consortium development in the planktonic and mineral attached cell subpopulations

**Figure 6:**
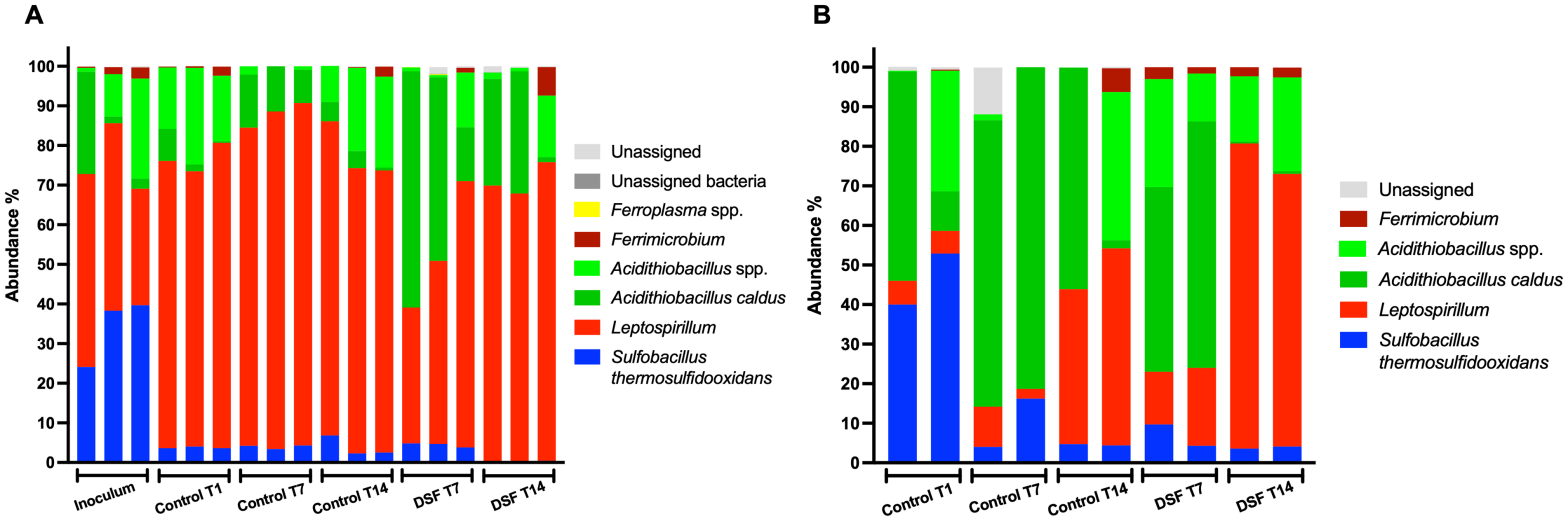
Effect of DSF/BDSF on community composition of MT consortium growth in pyrite. Relative abundances of assigned taxon in planktonic **(A)** and biofilm cell subpopulation **(B)** at 0 (inoculum), 1 (T1), 7 (T7), and 14-days (T14) are shown. DSF and BDSF were added at 2 μM each and incubated with 2% (wt/vol) pyrite at 40°C and 140 rpm agitation. Assays were triplicates (*n* = 3) or duplicates (*n* = 2) for planktonic and biofilm cell samples, respectively. Significant differences according to results of Kruskal–Wallis test are detailed in the results section.

The temporal development of biomass after 9 days and evaluation of mineral colonization at 1 and 9 days showed very low levels of planktonic and mineral-attached cells in DSF/BDSF treated samples. Nucleic acid extractions from samples taken at 1 day resulted in values below detection level, probably due to the small amount on biomass and the detrimental effect of DSF/BDSF on cell growth and/or viability. To obtain more biomass, especially from the mineral-attached samples, assays were conducted by incubating the cells with mineral for 24 h, prior to the addition of QS signal compounds (sampling time 1 day) and maintained for an additional 7 or 14 days, when cells were collected for nucleic acid extraction. The 16S rRNA gene amplicon data showed the intended inoculation ratio of 1:1:1 with the three species was partially met with an initial range of consortium composition of *S. thermosulfidooxidans* of 25–40%, *A. caldus* from 10–20%, and *L. ferriphilum* of 30–50% (Figure 6). During growth on 2% pyrite, the control MT consortium showed a significant increase in relative abundance of *L. ferriphilum* in the planktonic cell subpopulation (~70–80%; *p*-value between 0 and 7 days = 0.009). The increase in relative abundance displaced *S. thermosulfidooxidans* (~5%; *p*-value between 0 and 7 days = 0.004) while the *A. caldus* fraction remained constant (Figure 6A). The addition of DSF/BDSF showed a strong increase in the relative proportion of *A. caldus* (to ~45%) and a decrease in *L. ferriphilum* (to ~50%) in the planktonic cell subpopulation within 7 days as compared to the control consortium at the same time, where *L. ferriphilum* was in ~80% and *A. caldus* in ~15% (*p* = 0.028 and *p* = 0.024, respectively) (Figure 6A).

In the control biofilm cell subpopulation after 1 day, the culture was dominated by *A. caldus* and *S. thermosulfidooxidans* at ~46% each, while *L. ferriphilum* was only marginally present at 10–15%. As for the planktonic cell subpopulation, the *S. thermosulfidooxidans* relative abundance decreased with time (*p* = 0.023; Figure 6B) to approximately 5% and hence, *A. caldus* slightly dominated the pyrite-attached cell population. Furthermore, the addition of DSF/BDSF increased the relative abundance of *L. ferriphilum* in the biofilm subpopulation compared to the control cultures at 7 (*p* = 0.028) and 14 days (*p* = 0.008). In addition, *A. caldus* showed a significant reduction in DSF/BDSF treated samples after 14 days compared to the control cultures at the same time (*p* = 0.005; Figure 6B). Therefore, DSF/BDSF addition increased *L. ferriphilum* relative abundance in the biofilm subpopulation with a concomitant decrease in its planktonic fraction, meanwhile *A. caldus* had an opposite response with a decrease in its mineral-attached fraction and an increase in its planktonic cell subpopulation. In the case of *S. thermosulfidooxidans*, the presence of DSF/BDSF molecules did not affect its biofilm distribution at 7 and 14 days, while its planktonic subpopulation remained around 5% after 7 days and decreased to near zero at 14 days (Figures 6A,B).

**Figure 7:**
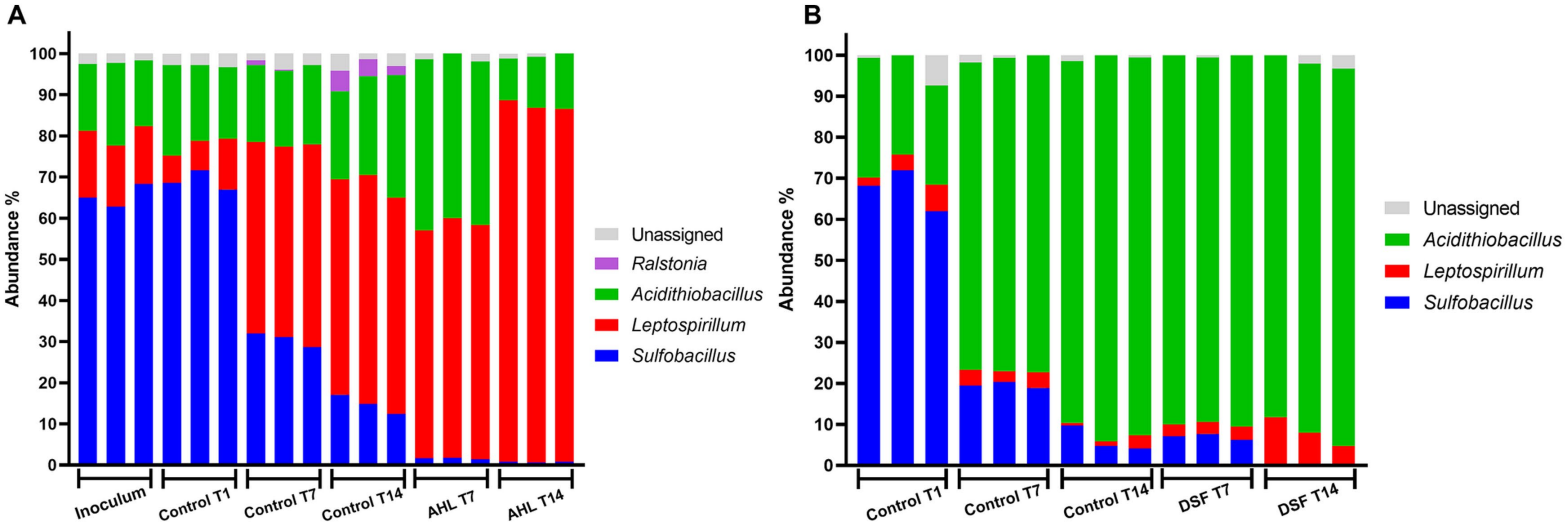
Effect of AHL mixture on community composition of MT consortium growth in pyrite. Relative abundances of assigned taxon in planktonic **(A)** and biofilm **(B)** cell subpopulations at 0 (inoculum), 1 (T1), 7 (T7), and 14-days (T14) are shown. AHLs (C12-AHL, C14-AHL, OH-C12-AHL, and OH-C14-AHL) were added at 5 μM each and incubated with 2% (wt/vol) pyrite at 40°C and 140 rpm agitation. Assays were triplicates (*n* = 3) and significant differences according to results of Kruskal–Wallis test are detailed in the results section.

To compare the effect of the AHL-based QS mechanism on the MT bioleaching consortium, similar assays were performed with addition of a mixture of AHLs instead of DSF/BDSF. In this study, the intended inoculation ratio of 1:1:1 was not met with an initial community composed of 65% *S. thermosulfidooxidans*, 20% *A. caldus*, and 15% *L. ferriphilum* (Figure 7). Control experiments showed a decrease in relative abundance in *S. thermosulfidooxidans* with time in both cell subpopulations, from ~70% to ~20% in planktonic fraction and from ~65% to ~10% in biofilm fraction (*p* < 0.0001 in both). While *L. ferriphilum* increased in planktonic cell relative abundance (*p* < 0.0001) but remained at very low levels in the biofilm (*p* = 0.22). *A. caldus* remained at a fraction of 17–19% of the planktonic cells during the first 7 days (*p* = 0.526) but then increased significantly to 25% between 7 and 14 days (*p* = 0.037). Similarly, the biofilm fraction of *A. caldus* increased from ~30% to ~90% between 1 and 14 days in control cultures (*p* < 0.0001). The addition of AHLs resulted in a fall from ~70% to ~2% of *S. thermosulfidooxidans* planktonic cell relative abundance after 7 and 14 days (*p* < 0.0001 & *p* = 0.0005, respectively). This correlated with an increase in *A. caldus* relative abundance to ~40% at 7 days (*p* < 0.0001), and 10% at 14 days (*p* = 0.078), while *L. ferriphilum* reached a relative abundance of ~55% at 7 days (*p* = 0.018), and ~87% at 14 days (*p* < 0.0001) (Figure 7A). Interestingly, pyrite-attached *S. thermosulfidooxidans* cells showed a lower proportion compared to the control (*p* < 0.0001 at 7 days, and *p* = 0.025 at 14 days) after AHL addition with a concomitant increase of *A. caldus* (*p* < 0.0001 at 7 days) that reached almost 90% relative abundance after 7 and 14 days post AHL addition. Finally, a small increase from ~2 to ~9% (*p* = 0.042 at 14 days) in *L. ferriphilum* was observed on day 14 (Figure 7B).

### 3.5 Effect of QS molecules on MT community RNA transcripts

**Figure 8:**
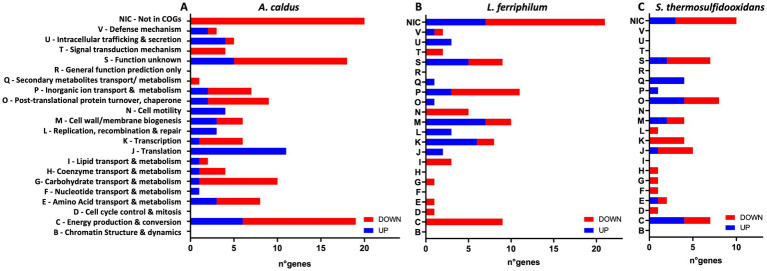
Effect of AHL mixture on RNA transcript changes in planktonic subpopulation of MT consortium growth in pyrite. Numbers of COG-assigned genes with statistically significant transcript numbers (*p*_adj_ <0.05) for *A. caldus* **(A)**, *L. ferriphilum* **(B)**, and *S. thermosulfidooxidans* **(C)**. Blue and red bars depict significant increased and decreased RNA transcripts, respectively. AHLs (C12-AHL, C14-AHL, OH-C12-AHL, and OH-C14-AHL) were added at 5 μM each after 24 h post inoculation and incubated with 2% (wt/vol) pyrite at 40°C and 140 rpm agitation for 14 days. Analyses were with four replicates (*n* = 4).

**Figure 9:**
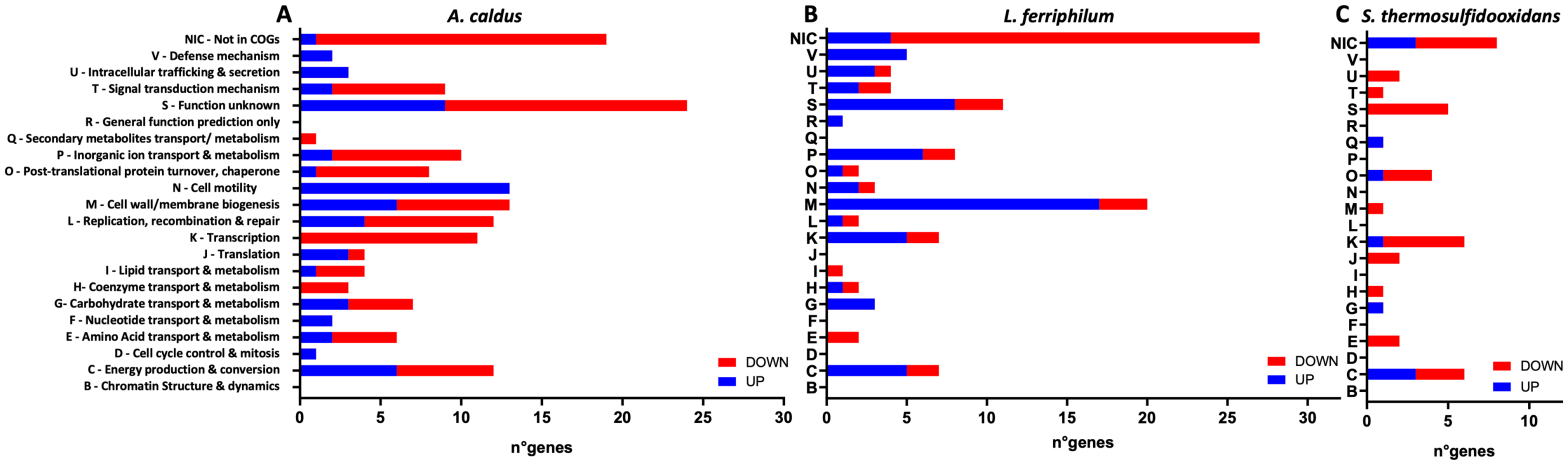
Effect of DSF/BDSF on transcriptional changes in planktonic subpopulation of MT consortium growth in pyrite. Numbers of COG-assigned genes with statistically significant transcript numbers (*p*_adj_ <0.05) for *A. caldus* **(A)**, *L. ferriphilum* **(B)**, and *S. thermosulfidooxidans* **(C)**. Blue and red bars depict significant increased and decreased RNA transcripts, respectively. DSF and BDSF were added at 2 μM each after 24 h post inoculation and incubated with 2% (wt/vol) pyrite at 40°C and 140 rpm agitation for 14 days. Analyses were with four replicates (*n* = 4).

The MT community planktonic subpopulation transcriptomic analyses after 14 days of AHLs or DSF/BDSF treatment showed significantly differing transcript numbers [|log_2_-fold change (LFC)| >1, FDR *p*-value (*p*_adj_) <0.05] according to the different conditions and species (Table 1, with full detail in Supplementary Table S1 for AHLs treatment and Supplementary Table S2 for DSF/BDSF treatment). To identify key regulated processes in each MT consortium member, these genes were grouped per species according to COG-categories (Figures 8, 9).

**Table 1:** RNA transcript number changes in cells of mock community after exposure to AHLs or DSF/BDSF.

QS treatment	Number of genes		*A. caldus*	*S. thermosulfidooxidans*	*L. ferriphilum*
AHL	Total	276	129	57	90
	Increased	103	45	22	36
	Decreased	173	84	35	54
DSF	Total	295	152	38	105
	Increased	126	55	8	63
	Decreased	169	97	30	42

Numbers of statistically significant RNA transcript numbers (|LFC| >1, *p*_adj_ <0.05) were grouped per condition and species.

#### 3.5.1 Effect of AHL-signal compounds on MT community RNA transcripts

AHL treatment on the MT community planktonic cells resulted in a total of 276 genes with significantly differing transcript numbers (103 increased and 173 decreased) distributed as 129, 90, and 57 for *A. caldus*, *L. ferriphilum*, and *S. thermosulfidooxidans*, respectively (Table 1). In descending order according to COG-categories (Table 2), 54 genes lacked a COG classification, 35 were involved in energy production and conversion processes (C category), 34 were of unknown function (S category), 20 were cell wall/membrane biogenesis (M category), 19 each in transcription and inorganic ion transport & metabolism (K & P categories), and 18 each for translation and post-translational protein turnover & chaperones (J & O categories). Similar profiles were evident when comparing the COG categories between species (Figure 8). However, the categories such as defense mechanism (V), intracellular trafficking & secretion (U), signal transduction mechanism (T), lipid transport & metabolism (I), and cell motility (N) only had increased transcripts in *A. caldus* and *L. ferriphilum*. Interestingly, all cell motility (N) genes with increased and decreased transcripts were assigned to *A. caldus* and *L. ferriphilum*, respectively suggesting an induction in motility in *A. caldus* and a repression in *L. ferriphilum*. Five of the nine differential RNA transcripts related to cell motility were directly related to flagellar machinery, most of them from *L. ferriphilum* (Supplementary Table S3). In this species, AHL treatment reduced transcript counts of four flagellar-related genes, including flagellar biosynthesis proteins FliR (LFTS_RS08685; LFC = −1.06) and FlhF (LFTS_RS08700; LFC = −2.06), in addition to flagellar structural proteins FliL (LFTS_RS08660; LFC = − 1.55), and a hook-basal body protein (LFTS_RS06585; LFC = −1.05). In contrast, the *A. caldus* gene encoding flagellin (ACAty_RS09530; LFC = 1.49) presented increased counts. In addition, *L. ferriphilum* showed a lower proportion of genes with increased transcript numbers related to post-translational protein turnover and chaperones (O category) compared to the other species, while a higher proportion of transcripts from genes related to secondary metabolites transport/metabolism (Q category) were increased in *S. thermosulfidooxidans*.

**Table 2:** Clusters of orthologous groups (COGs) analysis of RNA transcript number changes in cells of MT consortium after exposure to AHLs.

COG category	Total genes	Up	Down
B—Chromatin structure & dynamics	0	0	0
C—Energy production & conversion	35	10	25
D—Cell cycle control & mitosis	2	0	2
E—Amino acid transport & metabolism	11	4	7
F—Nucleotide transport & metabolism	2	1	1
G—Carbohydrate transport & metabolism	12	1	11
H—Coenzyme transport & metabolism	5	1	4
I—Lipid transport & metabolism	5	1	4
J—Translation	18	14	4
K—Transcription	19	7	12
L—Replication, recombination & repair	8	6	2
M—Cell wall/membrane biogenesis	20	12	8
N—Cell motility	9	4	5
O—Post-translational protein turnover, chaperone	18	7	11
P—Inorganic ion transport & metabolism	19	6	13
Q—Secondary metabolites transport/metabolism	6	5	1
R—General function prediction only	0	0	0
S—Function unknown	34	11	23
T—Signal transduction mechanism	6	0	6
U—Intracellular trafficking & secretion	8	7	1
V—Defense mechanism	5	3	2
NIC—Not in COGs	54	10	44

Numbers of COG-assigned genes with statistically significant RNA transcript numbers with *p*_adj_ <0.05 are grouped according to LFC values as significant increased (LFC >1) and significant decreased genes (LFC <1).

#### 3.5.2 Effect of DSF-signal compounds on the MT community RNA transcripts

The addition of the mixtures of DSF/BDSF to the MT community showed a similar profile of genes with differing transcript counts compared as AHL treatment (Table 1). A total of 295 genes exhibited significantly differing transcript numbers (126 and 169 were increased and decreased, respectively) that were distributed as 152, 105, and 38 for *A. caldus*, *L. ferriphilum*, and *S. thermosulfidooxidans*, respectively. *A. caldus* and *S. thermosulfidooxidans* showed fewer genes with increased counts while *L. ferriphilum* showed a higher proportion of increased RNA transcripts (Table 1). Analysis of the COG categories (Table 3) showed an important effect in cell wall/membrane biogenesis (M category; 34 genes), cell energy production & conversion (C category; 25 genes), and transcription (K category; 24 genes). Once again, genes with unknown function (S category) or without a classification (Not in COGs) showed a high number of altered transcripts with 40 and 54 genes, respectively (Table 3). When comparing the COG categories between species (Figure 9), similar profiles were described for *A. caldus* and *L. ferriphilum*. Once again, some categories such as defense mechanisms (V), post-translational protein turnover & chaperones (P), replication & cell motility (N), recombination & repair (L), and lipid transport & metabolism (I) only presented changes in *A. caldus* and *L. ferriphilum*. Interestingly, DSF/BDSF treatment produced an opposite effect compared to AHLs in genes related to cell motility (N category) of *A. caldus*, whose transcript counts increased.

**Table 3:** Clusters of orthologous groups (COGs) analysis of RNA transcript number changes in cells of mock community after exposure to DSF/BDSF.

COG category	Total genes	Up	Down
B—Chromatin structure & dynamics	0	0	0
C—Energy production & conversion	25	14	11
D—Cell cycle control & mitosis	1	1	0
E—Amino acid transport & metabolism	10	2	8
F—Nucleotide transport & metabolism	2	2	0
G—Carbohydrate transport & metabolism	11	7	4
H—Coenzyme transport & metabolism	6	1	5
I—Lipid transport & metabolism	5	1	4
J—Translation	6	3	3
K—Transcription	24	6	18
L—Replication, recombination & repair	14	5	9
M—Cell wall/membrane biogenesis	34	23	11
N—Cell motility	16	15	1
O—Post-translational protein turnover, chaperone	14	3	11
P—Inorganic ion transport & metabolism	18	8	10
Q—Secondary metabolites transport/metabolism	2	1	1
R—General function prediction only	1	1	0
S—Function unknown	40	17	23
T—Signal transduction mechanism	14	4	10
U—Intracellular trafficking & secretion	9	6	3
V—Defense mechanism	7	7	0
NIC—Not in COGs	54	8	46

Numbers COG-assigned differentially expressed genes with *p*_adj_ <0.05 are grouped according to LFC values as significant increased (LFC >1) and significant decreased genes (LFC <1).

Notably, *L. ferriphilum* exhibited a high proportion of genes with increased counts related to cell wall/membrane biogenesis (M). Part of this response may be involved with an induction in biosynthesis of membrane components related to surface attachment and may explain the increased proportion of *L. ferriphilum* in the biofilm according to the 16S rRNA gene sequencing (Figure 6). Detailed analysis of genes related to cell motility (N) revealed DSF/BDSF treatment resulted in increased transcript counts from 15 genes and a decrease in one gene. Most of the induced genes encoded *A. caldus* flagellar proteins including FliD (ACAty_RS09525; LFC = 6.64), flagellin (ACAty_RS09530, LFC = 4.05; ACAty_RS06155, LFC = 1.89), FlhA (ACAty_RS09435; LFC = 2.41), FliM (ACAty_RS06230; LFC = 2.02), and four flagellar hook-associated proteins (ACAty_RS09555, LFC = 3.04; ACAty_RS09550, LFC = 2.14; ACAty_RS06140, LFC = 1.92; ACAty_RS06145, LFC = 1.3) (Supplementary Table S3). Overall, the results strongly suggested that DSF/BDSF treatment induced flagellar machinery in *A. caldus* while in *L. ferriphilum* a likely induction in biosynthesis of membrane components related to surface attachment may increase its colonization capacity. In contrast, *S. thermosulfidooxidans* presented a general reduction of RNA counts related to intracellular trafficking & secretion (U) and signal transduction mechanisms (T). It is important to note that *S. thermosulfidooxidans* showed a smaller magnitude in gene transcript changes compared to the other two species, suggesting this bacterium was less affected by both AHL or DSF/BDSF treatments.

## 4 Discussion

### 4.1 DSF/BDSF effect on the redox potential in pyrite and chalcopyrite cultures

The ORP in acidic solutions such as acid mine drainage (AMD) and bioleaching liquors is controlled by the ratio of dissolved iron(II) and iron(III) ions. This is a key parameter for optimizing chalcopyrite bioleaching (Hiroyoshi et al., 2008), which is optimal in the range of 420–550 mV [Ag/AgCl]. Chalcopyrite concentrates bioleached with weak iron-oxidizers instead of the strong iron-oxidizer *L. ferriphilum* maintains the ORP within the desired range that results in improved copper release (Christel et al., 2018). Considering that *L. ferriphilum* is one of the predominant species in natural leaching environments, its DSF/BDSF production may be used to inhibit the iron oxidizing activity of its neighboring species (Bellenberg et al., 2018, 2021). Although the addition of DSF/BDSF resulted in lower ORP levels with the MT community, chalcopyrite bioleaching was not improved (Figure 4D). This was probably due to detrimental effects on growth, colonization, and/or iron oxidation in *L. ferriphilum* and *S. thermosulfidooxidans*. The inhibitory effect on *L. ferriphilum* and *S. thermosulfidooxidans* growth was demonstrated in pyrite cultures (Figure 1A) and it has been proven that DSF and BDSF treatment inhibits chalcopyrite oxidation in axenic cultures of the three MT consortium members during a cultivation period of 32 days (Bellenberg et al., 2018). Here we demonstrated the same effect on the oxidation capacities of *L. ferriphilum* and *S. thermosulfidooxidans* growth in pyrite for 9 days (Figure 1A) and in mixed cultures of the three species on chalcopyrite over the same period (Figure 4B). Therefore, copper recovery was not improved as the treatment similarly affected the two iron-oxidizing members such that the ORP values were below the optimal window for chalcopyrite bioleaching. Importantly, the reduction of the iron oxidation activity upon DSF/BDSF addition was only observed at 3 days for the mixed culture on pyrite (Figure 1B). This suggested the MT consortia iron oxidation capacity with pyrite as electron donor was less sensitive to DSF/BDSF compared to axenic cultures of the individual species. This differential effect was also observed for mineral colonization as DSF/BDSF presence hindered MT attachment to chalcopyrite for a longer period (Figure 5) than was the case for pyrite (Figure 3).

### 4.2 Dual effect of QS molecules on the MT consortium

EFM analysis, 16S rRNA gene-based relative abundances, and RNA transcript changes all suggested DSF/BDSF induced two responses by the MT consortium: one related to the activation of the QS signaling pathways with changes in EPS biosynthesis and cell dispersion (discussed below) along with a second response related to activation of detoxification mechanisms due to the detrimental effect of high concentrations of fatty acids on cells. A harmful effect of DSF/BDSF on cell viability was suggested in the MT consortium 16S rRNA gene sequencing cultured with chalcopyrite, which resulted in extremely low biomass and low-quality DNA obtained from the mineral fractions and while the planktonic population could be analyzed, no significant changes were observed (data not shown). Therefore, the DSF/BDSF treatment likely resulted in some cell death within the biofilm subpopulation due to the low or absent iron oxidation and resulting in biomass recovery from the mineral samples being challenging. In addition, the EFM results suggested that the MT consortium control culture mineral colonization was significantly lower in chalcopyrite compared to pyrite and thus, it was unlikely to be possible to recover sufficient biomass for DNA extraction from biofilm samples.

In addition to a degree of cell death, DSF-family compounds may induce biofilm dispersal (Figure 5), as described for other bacterial models such as *Xanthomonas campestris* and *Pseudomonas aeruginosa* (Ryan et al., 2015). This effect was at least shown to occur for *A. caldus* in MT consortium in pyrite grown cultures. The EFM results showed that MT consortium colony density levels and areas of colonies on pyrite were not affected by DSF/BDSF treatment at 9 days (Figure 3). In contrast, 16S rRNA gene-based community analyses of these samples demonstrated that after DSF treatment, *L. ferriphilum* increased in relative abundance in the mineral-attached subpopulation and there was a concomitant decrease in *A. caldus* (Figure 6B) while the opposite behavior was detected in the planktonic cell subpopulation (Figure 6A). This strongly suggested that DSF/BDSF promotes displacement of *A. caldus* and further mineral colonization by *L. ferriphilum* on pyrite. The DSF/BDSF dispersal effect on pyrite attached *A. caldus* was supported by the transcriptomic analyses where transcript counts suggested an induction of the flagellar machinery as part of a transition from biofilm to planktonic lifestyle. It is important to note that DSF/BDSF effects on *L. ferriphilum* and *A. caldus* colonization capacities depend on the presence of the other species in the mineral grown culture. This was supported by the EFM microscopy data from axenic cultures on pyrite that showed DSF/BDSF treatment reduced *L. ferriphilum* and increased *A. caldus* colonization after 9 days (Figure 2B) that contrasted with the 16S rRNA gene-based results where both species were present in the MT consortium.

### 4.3 Effect of QS compounds on *Leptospirillum ferriphilum* RNA transcripts

RNA transcript counts for genes annotated to *L. ferriphilum* obtained from the MT consortium planktonic cells were compared to a previous study using axenic cultures of *L. ferriphilum* iron(II)-grown cells (Bellenberg et al., 2021). The comparison showed groups of 18 and 14 shared genes for the DSF and AHLs treatments, respectively. Three of the *L. ferriphilum* genes with increased RNA transcript counts were common for AHLs and DSF treatments, which suggested they were part of a similar QS response regulation system (Supplementary Tables S4, S5). Two of these genes encoded proteins annotated as components of efflux transporter systems: efflux transporter outer membrane subunit (LFTS_RS05130, COG category M) and multidrug efflux RND transporter permease subunit (LFTS_RS05135, COG category V). In addition, the HlyD family secretion protein (LFTS_RS12470, COG category V) and the TetR/AcrR family transcriptional regulator (LFTS_RS12460, COG category K) also had increased counts in DSF/BDSF treated cells. TetR-family transcriptional regulators (TFTRs) are DNA binding factors that regulate expression of a wide range of genes, including efflux pumps (Johnson and Shafer, 2015; Colclough et al., 2019). Likely, the TetR/AcrR family transcriptional regulator mediated the increased transcript counts of the three (and potentially other) proteins related to bacterial efflux pumps in *L. ferriphilum*. In Gram-negative bacteria, most efflux transporter systems, such as RND (Resistance-nodulation-division), MFS (Major facilitator superfamily), and ABC (ATP-binding cassette) pumps form a tripartite system to extrude substances directly from cytoplasm to the extracellular environment. These pumps consist of an inner membrane protein (IMP), an outer membrane protein (OMP), and a membrane fusion protein (MFP) to link the IMP and the OMP (Klenotic et al., 2021). The mentioned multidrug efflux RND transporter permease subunit (LFTS_RS05135) was identified as an IMP and the efflux transporter outer membrane subunit (LFTS_RS05130) as an OMP that are both part of the same operon probably regulated by TetR/AcrR family transcriptional regulator. The MFP component and the RND transporter periplasmic adaptor subunit (LFTS_RS05140) encoded in the same operon that was not detected in the previous transcriptomic analysis (Bellenberg et al., 2021), also showed increased transcripts counts under both QS treatments in this study. Furthermore, the HlyD family secretion protein (LFTS_RS12470) has been identified as a homolog of *Burkholderia cepacia* fusaric acid resistance protein (FusE). In *B. cepacian*, FusE is an efflux pump involved in the resistance of the fungal toxin fusaric acid (Mohd Din and Othman, 2023), an antimicrobial that causes membrane disruption in Gram-positive bacteria (Parsons et al., 2012). Fusaric acid is an alpha, beta-unsaturated monocarboxylic acid and therefore presents a similar chemical nature compared to DSF/BDSF. This could explain the increased transcripts of this detoxification system as a response to the high concentration of DSF/BDSF. Overall, this response suggested a detrimental effect of both AHLs and DSF/BDSF treatments. Upon exposure to QS molecules, cells may activate efflux pumps to export these detrimental compounds. These results support a previous *L. ferriphilum* transcriptional analysis of DSF addition (Bellenberg et al., 2021) where it was proposed that the DSF transcriptional effect not only involves a response via DSF-response regulators but also included a response to fatty acid stress upon direct uptake of DSF/BDSF molecules. It also cannot be ruled out that this extrusion is part of a response to “turn off” the QS signaling, but to the best of our knowledge this has not been described.

Comparison between genes regulated by DSF/BDSF addition in the previous transcriptomic analysis and the set of genes described here identified three genes that encode proteins related to EPS synthesis (Supplementary Table S4). This result indicated the production of some EPS components. For instance, glutamine-fructose-6-phosphate transaminase (GFPT, LFTS_RS10570, logFC = 3.81, COG category M) and UDP-3-O-acyl-N-acetylglucosamine deacetylase (LFTS_RS07285, logFC = −1.09, COG category M) are involved in hexosamine and lipopolysaccharide (LPS) biosynthesis. GFPT produces D-glucosamine-6-phosphate (GlcN-6-P), which is an essential precursor of hexosamines, such as uridine 5′-diphosphate-N-acetyl-D-glucosamine (UDP-GlcNAc). UDP-GlcNAc is a substrate for the synthesis of glycoproteins, proteoglycans, and glycolipids including EPS components (Yamazaki, 2014). Lipopolysaccharide (LPS) is one of the glycolipids depending on UDP-GlcNAc production as the lipid A portion of LPS is synthetized from this molecule. One key enzyme in this biosynthesis pathway is UDP-3-O-acyl-N-acetylglucosamine deacetylase (LpxC), which catalyzes the second reaction in lipid A biosynthesis pathway (Niu et al., 2023). Therefore, the increase in GFPT transcripts and the decreased transcripts of LpxC described here suggests an activation of the hexosamine biosynthesis pathway that may result in increased production of some EPS components (different from LPS) such as glycoproteins, proteoglycans, and/or glycolipids. The third EPS-related gene encodes the TIGR03013 family PEP-CTERM/XrtA system glycosyltransferase (LFTS_RS10450, logFC = 2.82, COG category M). This glycosyltransferase shows homology with the *Vibrio cholerae* exopolysaccharide biosynthesis glycosyltransferase (VpsL) and *Methylobacillus* sp. (EpsB) (identity ~30%/similarity ~48%). Both enzymes catalyze the transfer of the glucose moiety to the corresponding EPS structure in the respective bacterium (VPS polysaccharide in *V. cholerae* and methanolan in *Methylobacillus* sp.) (Fong et al., 2010; Yoshida et al., 2003). Therefore, the LFTS_RS10450 gene should encode a similar glycosyltransferase responsible for biosynthesis of some *L. ferriphilum* EPS and its increased transcripts may lead to EPS production.

In the case of the comparison between genes regulated by AHLs molecules, two genes encoding proteins related to flagellar machinery and three related to nitrogen metabolism had decreased RNA transcripts (Supplementary Table S5). As mentioned in the results section, AHLs induced changes in transcript counts of some *L. ferriphilum* flagellar genes. Accordingly, two of these genes were previously detected in a transcriptomic analysis performed in *L. ferriphilum* (Bellenberg et al., 2021). In this bacterium, AHLs may induce a deregulation of flagellar rotational direction, since genes coding for FliL (LFTS_RS08660, LFC = −1.55, COG category N) and FliR (LFTS_RS08685, LFC = −1.06, COG category N) had decreased RNA counts after AHLs addition. The flagellar basal body-associated FliL family protein directly controls the rotational direction of flagella during chemotaxis, while FliR is a flagellar export pore protein required for formation of the rod structure of the flagellar apparatus (Takekawa et al., 2019). This deregulation could explain the increased 16S rRNA gene-based presence of *L. ferriphilum* in the biofilm subpopulation due to a lower motility, in accordance with a sessile lifestyle.

Another group of genes with decreased RNA transcripts was related to nitrate metabolism. Two of these genes code for nitrogen regulatory proteins; the P-II family nitrogen regulator (LFTS_RS00615, LFC = −2.63, COG category K) and the molybdopterin-dependent oxidoreductase (LFTS_RS00620, LFC = −2.64 COG category V). The first regulator indirectly controls the glutamine synthetase gene (*gln*A) transcription (Son and Rhee, 1987). Meanwhile, a conserved domain analysis showed that the molybdopterin-dependent oxidoreductase protein possesses two domains, one regulator domain (annotated as GlnK) and a nitrate reductase domain. In bacteria, nitrate reductases are described as essential for nitrate/nitrite assimilation (Goddard et al., 2008) and therefore, AHLs may suppress *L. ferriphilum* nitrogen incorporation.

### 4.4 Effects of QS treatment on the MT *rpf* cluster expression and regulation of c-di-GMP levels

AHL addition caused a decrease in MT consortium DSF-related genes RNA transcript counts including *L. ferriphilum* DSF-synthase (RpfF homolog; LFTS_RS10260, LFC = −1.05) and two putative *A. caldus* cytoplasmatic DSF-receptors (RpfR homologous; ACAty_RS13840, LFC = −1.65; ACAty_RS08360, LFC = −1.04) (Supplementary Table S1). Importantly, a gene encoding a diguanylate cyclase (DGC) had decreased counts in response to AHL exposure (ACAty_RS14845 LFC = −1.12), which may suggest AHL exposure reduced the *A. caldus* cyclic diguanylate (c-di-GMP) levels. This DGC is related to regulation of *A. caldus* swarming motility and adherence to sulfur surfaces (Castro et al., 2015). Overall, this potential reduction in c-di-GMP levels could induce a transition from a sessile to a planktonic lifestyle in *A. caldus* and explain the increased level of this species in the pyrite attached subpopulation (Figure 7).

DSF/BDSF treatment induced a similar profile of transcript changes in *L. ferriphilum* DSF-related genes. For instance, DSF-synthase (LFTS_RS10260, LFC = −1.31) showed a similar decrease in counts in addition to three putative RpfR receptors, one from *L. ferriphilum* (LFTS_RS04005, LFC = − 1.81) and two from *A. caldus* (ACAty_RS13840, LFC = − 1.31; ACAty_RS08360, logFC = − 1.76) (Supplementary Table S2). Furthermore, two genes encoding *L. ferriphilum* c-di-GMP receptors had increased counts (LFTS_RS11415, LFC = 1.57; LFTS_RS06385, LFC = 1.53) while one had lower counts in *A. caldus* (ACAty_RS06175, LFC = −1.25). These proteins possess a PilZ domain, which binds c-di-GMP to exert a response to this second messenger through other effector domains. In the case of *L. ferriphilum*, the other domains possess a glycosyltransferase activity that may mediate a response to DSF/BDSF through changes in the synthesis of some oligosaccharides, polysaccharides, and/or glycoconjugates. Therefore, the transcriptome analyses suggested negative feedback exerted by QS molecules in DSF-signaling, as described for this communication system in several Gram-negative bacteria as a “turn off” mechanism (He et al., 2023). Finally, no *S. thermosulfidooxidans* DSF related genes were identified with significant changes in transcript counts.

## 5 Conclusion

This study evaluated if DSF/BDSF treatment differentially affected *L. ferriphilum* iron oxidizing and/or colonization capacity, in order to reduce the presence of a strong iron oxidizer and maintain ORP values in the range required for optimal chalcopyrite lixiviation. Accordingly, it was observed that DSF/BDSF molecules prevented pyrite colonization by *L. ferriphilum* for longer periods (1 to 9 days) compared to *A. caldus* and *S. thermosulfidooxidans*, whose colonization was partially affected at 1 and 9 days, respectively. However, this effect was different when bacteria were exposed to DSF/BDSF as a consortium, since DSF/BDSF induced a displacement of *A. caldus* from pyrite by *L. ferriphilum*, concomitantly with a drop in ORP values below the optimal range for copper dissolution from the mineral. Therefore, DSF/BDSF treatment did not improve pyrite and chalcopyrite bioleaching, as observed in assays with the MT consortium cultured in the presence of these two minerals. Furthermore, the MT consortium grown on pyrite was less sensitive to DSF/BDSF addition compared to axenic cultures, with a reduction of iron oxidation only at 3 days, a decrease in their cell density after only the first day of treatment, and better pyrite colonization at 9 days. Unfortunately, this resistance was not observed in chalcopyrite assays, since DSF/BDSF addition prevented bacterial colonization and resulted in a lowered mineral dissolution. This stronger result was probably due to a mixed effect that included lower iron oxidation and mineral colonization added to a certain degree of cell death in especially mineral-attached cells of *L. ferriphilum* and *S. thermosulfidooxidans*. Therefore, this study demonstrated that QS treatment changed the distribution of planktonic/mineral subpopulations of the three MT consortium species. In addition to DSF/BDSF effects, AHL addition correlated with a decrease in the relative abundance of *S. thermosulfidooxidans* in planktonic and biofilm subpopulations with an increase of *L. ferriphilum* and *A. caldus* in the planktonic subpopulation and solely *A. caldus* in the biofilm. Transcriptomic analyses showed a complex cellular response to QS molecules in *L. ferriphilum* and *A. caldus* with one response related to activation of the QS signaling pathways, including decreased RNA counts for *L. ferriphilum* DSF synthase and a DGC in *A. caldus* in addition to other c-di GMP related proteins, EPS synthesis, and flagellar-associated proteins. DSF signaling also induced *A. caldus* motility while repressing the *L. ferriphilum* flagellar machinery. This was in accordance with microscopy results obtained from axenic cultures and the 16S rRNA gene-based analyses performed on the MT consortium grown on pyrite. Finally, the second QS response was related to the detrimental effect of high concentrations of fatty acids on cells with activation of detoxification mechanisms. In summary, this study provided insights into the manipulation of bioleaching consortia to promote or prevent the presence of specific species and/or metabolic activities. Treatments emulating DSF/BDSF effects on mineral colonization and mineral lixiviation may be designed to prevent the uncontrolled leaching process resulting in acid mine drainage and other similar environmental problems.

## Data Availability

The raw sequencing data for the twelve MT consortium samples were deposited in NCBI Sequence Read Archive (SRA) under accession SAMN48368890 to SAMN48368901, associated with BioProject PRJNA1259591.
