# Supplementary material for: QS molecules change the planktonic/mineral subpopulations distribution of moderately thermophilic leaching bacteria in pyrite and decrease leaching in chalcopyrite
Source: Front Microbiol. 2025 May 16;16:1592588. doi: 10.3389/fmicb.2025.1592588 (PMC12122527; doi:10.3389/fmicb.2025.1592588)
Supplement: Supplementary file 2 [file Data_Sheet_1.docx]

Supplementary Material

**A**

**Figure S1.** Relative abundance of RNA transcript counts expressed as percent of total counts per replicate. Percent is presented per species of the MT consortia; *L. ferriphilum* (red), *At. caldus* (green), and *S. thermosulfidooxidans* (blue) for each condition; control (C1 – C4), AHLs-treated (AHL1 – AHL4), and DSF/BDSF-treated (DSF1 – DSF 4). Analyzes Analyses were with four replicates (*n*=4).

**B**

**C**

**Figure S2.** Coefficient of variation according to the number of EFM images used to determine colonization parameters of axenic cultures of acidophilic leaching bacteria at 1 day (A), 9 days (B), and the MT consortium at 1 and 9 days (C) on the mineral surface. Axenic or mixed cultures of *L. ferriphilum* (L), *At. caldus* (C), and *S. thermosulfidooxidans* (S) were prepared in duplicates (Rep1 and Rep2). DSF and BDSF were added at 2 µM each and incubated with 2% (wt/vol) **pyrite**, at 40 °C with agitation at 140 rpm.

**Figure S3.** Coefficient of variation according to the number of EFM images used to determine colonization parameters of the MT consortium at 1 and 9 days (C) on the mineral surface. Mixed cultures of *L. ferriphilum* (L), *At caldus* (A), and *S. thermosulfidooxidans* (S) were prepared in duplicates (Rep1 and Rep2). DSF and BDSF were added at 2 µM each and incubated with 2% (wt/vol) **chalcopyrite,** at 40 °C with agitation at 140 rpm.

**Table S3. Effect of treatment with quorum sensing molecules in flagellar machinery of the MT consortia.** AHLs treatment represses flagellar machinery in *L. ferriphilum* while DSF/BDSF induces flagellar machinery in *At. caldus.* Data are shown as a list of genes with significant changes in their transcript levels (|LFC| > 1, padj < 0.05) determined by DESeq2 analysis of raw counts of RNASeq data from four replicates (*n*=4).

| AHLs treatment | | | | |
| --- | --- | --- | --- | --- |
| locus tag | **LFC** | **gene name** | **Description** | **NCBI Reference Sequence** |
| ACAty_RS09530 | 1.491 | *-* | Flagellin, protein which polymerizes to form the filaments of bacterial flagella. | WP_004873035.1 |
| LFTS_RS06585 | -1.051 | *-* | Flagellar basal body rod FlgEFG protein C-terminal, is flagellar hook-basal body protein. | WP_036080728.1 |
| LFTS_RS08685 | -1.062 | *fliR* | Bacterial export proteins, family 1. Flagellar export pore protein with a role in flagellar biosynthesis. Belongs to the FliR/MopE/SpaR family. | WP_023524376.1 |
| LFTS_RS08660 | -1.552 | *fliL* | Flagellum-specific ATP synthase. Controls the rotational direction of flagella during chemotaxis. | WP_014960005.1 |
| LFTS_RS08700 | -2.057 | *-* | Flagellar biosynthesis protein FlhF, is a SRP54-type protein with a GTPase domain. | WP_023524373.1 |
|  |  |  |  |  |
| DSF/BDSF treatment | | | | |
| ACAty_RS09525 | 6.64 | *fliD* | Required for morphogenesis and for the elongation of the flagellar filament by facilitating polymerization of the flagellin monomers at the tip of growing filament. | WP_004873034.1 |
| ACAty_RS09530 | 4.05 | *-* | Flagellin, protein which polymerizes to form the filaments of bacterial flagella. | WP_004873035.1 |
| ACAty_RS09555 | 3.04 | *flgK* | Flagellar hook-associated protein with flagellar basal body rod FlgEFG protein C-terminal. | WP_004873040.1 |
| ACAty_RS09435 | 2.41 | *flhA* | Flagellar biosynthesis protein FlhA. Required for formation of the rod structure of the flagellar apparatus. Together with FliI and FliH, may constitute the export apparatus of flagellin. | WP_051620864.1 |
| ACAty_RS09550 | 2.14 | *flgL* | Flagellar hook-associated protein with flagellin N-terminal helical region. | WP_004873038.1 |
| ACAty_RS06230 | 2.02 | *-* | Flagellar motor switch protein FliM. | WP_004871987.1 |
| ACAty_RS06140 | 1.92 | *-* | Flagellar hook-associated protein with flagellar basal body rod FlgEFG protein C-terminal. | WP_004871969.1 |
| ACAty_RS06155 | 1.89 | *-* | Flagellin-like protein. Protein which polymerizes to form the filaments of bacterial flagella. | WP_004871972.1 |
| ACAty_RS06145 | 1.30 | *-* | Flagellar hook-associated protein with flagellin N-terminal helical region. | WP_004871970.1 |
| ACAty_RS06015 | 2.26 | *-* | Chemotaxis sensory transducer. Methyl-accepting chemotaxis protein (MCP), mediates chemotaxis by altering swimming behavior | WP_004871929.1 |
| ACAty_RS06035 | 1.67 | *-* | Chemotaxis protein CheA, is a sensor histidine protein kinase that transmits sensory signals from chemoreceptors to the flagellar motors | WP_004871938.1 |
| ACAty_RS05890 | 3.79 | *-* | PilW family protein, similar to type IV pilus (T4P) assembly protein PilW. T4P performs functions such as locomotion, adherence to host cells, DNA uptake, protein secretion, etc. | WP_004871861.1 |
| ACAty_RS05900 | 1.67 | *-* | Tfp pilus assembly protein tip-associated adhesin PilY1, belong to FhaB superfamily | WP_004871866.1 |
| LFTS_RS10360 | 2.55 | *-* | Bacterial-type flagellum-dependent cell motility with DUF4091 domain with unknown function | WP_036081514.1 |
| LFTS_RS08940 | -1.05 | *-* | tetratricopeptide repeat protein | WP_023524333.1 |

**Table S4.** **Statistically significant RNA transcript numbers for iron(II)-grown cells of *Leptospirillum ferriphilu*m DSM 14647 axenic culture (Bellenberg *et al*., 2021) and *Leptospirillum ferriphilu*m DSM 14647 in MT consortia (this study).** Data are from cells after exposure to 2 µM **DSF** and 2 µM **BDSF,** showed as a list of genes with significant changes in their transcript levels (|LFC| > 1, *padj* < 0.05) determined by DESeq2 analysis of raw counts of RNASeq data from four replicates (*n*=4).

| locus tag | LFC | *padj* | gene name | annotation | NCBI Reference Sequence |
| --- | --- | --- | --- | --- | --- |
| LFTS_RS10570 | 3.81 | 1.5.E-135 | glmS | glutamine-fructose-6-phosphate transaminase (isomerizing) | WP_036081614.1 |
| LFTS_RS10445 | 3.09 | 1.1.E-59 |  | SLBB domain-containing protein | WP_036081550.1 |
| LFTS_RS10450 | 2.82 | 1.9.E-83 |  | TIGR03013 family PEP-CTERM/XrtA system glycosyltransferase | WP_036081553.1 |
| LFTS_RS10285 | 2.53 | 1.2.E-37 |  | cistern family PEP-CTERM protein | WP_052157770.1 |
| LFTS_RS10400 | 2.18 | 4.1.E-39 |  | glycosyltransferase family 2 protein | WP_052157775.1 |
| LFTS_RS05130 | 1.85 | 1.3.E-103 |  | efflux transporter outer membrane subunit | WP_014961731.1 |
| LFTS_RS05135 | 1.84 | 8.1.E-83 |  | multidrug efflux RND transporter permease subunit | WP_036081968.1 |
| LFTS_RS12460 | 1.78 | 3.0.E-86 |  | TetR/AcrR family transcriptional regulator | WP_036082305.1 |
| LFTS_RS10395 | 1.63 | 3.7.E-25 |  | glycosyltransferase family 2 protein | WP_036081527.1 |
| LFTS_RS12465 | 1.54 | 1.4.E-84 |  | cytochrome c family protein | WP_036082306.1 |
| LFTS_RS10540 | 1.52 | 5.4.E-36 |  | NAD-dependent epimerase | WP_036081600.1 |
| LFTS_RS12470 | 1.48 | 2.3.E-123 |  | HlyD family secretion protein | WP_099590644.1 |
| LFTS_RS10605 | 1.38 | 2.3.E-15 |  | PEP-CTERM sorting domain-containing protein | WP_036081628.1 |
| LFTS_RS10195 | 1.18 | 7.2.E-11 |  | hypothetical protein | WP_143469085.1 |
| LFTS_RS08940 | -1.05 | 4.3.E-52 |  | tetratricopeptide repeat protein | WP_023524333.1 |
| LFTS_RS07285 | -1.09 | 1.6.E-28 | lpxC | UDP-3-O-acyl-N-acetylglucosamine deacetylase | WP_036081051.1 |
| LFTS_RS02665 | -1.14 | 7.9.E-23 |  | hypothetical protein | WP_014961248.1 |
| LFTS_RS04000 | -1.64 | 7.5.E-45 |  | GGDEF domain-containing protein | WP_049713715.1 |

**Table S5.** **Statistically significant RNA transcript numbers for iron(II)-grown cells of *Leptospirillum ferriphilu*m DSM 14647 axenic culture (Bellenberg *et al*., 2021) and *Leptospirillum ferriphilu*m DSM 14647 in in MT consortia (this study).** Data are from cells after exposure to AHLs (C12-AHL, C14-AHL, OH-C12-AHL, and OH-C14-AHL; 2 µM each) shown as a list of genes with significant changes in their transcript levels (|LFC| > 1, *padj* < 0.05) determined by DESeq2 analysis of raw counts of RNASeq data from four replicates (*n*=4).

| locus tag | LFC | *padj* | gene name | annotation | NCBI Reference Sequence |
| --- | --- | --- | --- | --- | --- |
| LFTS_RS05130 | 3.83 | 0.0.E+00 | - | efflux transporter outer membrane subunit | WP_014961731.1 |
| LFTS_RS05135 | 3.09 | 1.1.E-258 | - | multidrug efflux RND transporter permease subunit | WP_036081968.1 |
| LFTS_RS06085 | 1.28 | 2.4.E-21 | - | Hsp20/alpha crystallin family protein | WP_023524701.1 |
| LFTS_RS12460 | 1.04 | 4.2.E-27 | - | TetR/AcrR family transcriptional regulator | WP_036082305.1 |
| LFTS_RS10260 | -1.05 | 9.7.E-28 | - | Enoyl-CoA hydratase/isomerase | WP_036081469.1 |
| LFTS_RS06585 | -1.05 | 2.3.E-01 | - | Flagellar basal body rod FlgEFG protein C-terminal | WP_036080728.1 |
| LFTS_RS08685 | -1.06 | 1.8.E-01 | fliR | Bacterial export proteins, family 1 | WP_023524376.1 |
| LFTS_RS08590 | -1.14 | 1.4.E-03 | flrB | phosphorelay sensor kinase activity | WP_023524390.1 |
| LFTS_RS08705 | -1.25 | 9.0.E-02 | - | Cellulose biosynthesis protein BcsQ | WP_036080049.1 |
| LFTS_RS09180 | -1.27 | 1.2.E-02 | - | Nitrogen regulatory protein P-II | WP_023524249.1 |
| LFTS_RS08660 | -1.55 | 7.2.E-06 | fliL | flagellar basal body-associated FliL family protein | WP_014960005.1 |
| LFTS_RS08585 | -1.88 | 3.5.E-09 |  | tetratricopeptide repeat protein | WP_036080014.1 |
| LFTS_RS00615 | -2.63 | 1.8.E-05 | - | P-II family nitrogen regulator | WP_099590502.1 |
| LFTS_RS00620 | -2.64 | 1.1.E-20 | - | molybdopterin-dependent oxidoreductase | WP_180271610.1 |
